# A lightweight attention deep learning method for human-vehicle recognition based on wireless sensing technology

**DOI:** 10.3389/fnins.2023.1135986

**Published:** 2023-02-09

**Authors:** Mingxin Song, Rensheng Zhu, Xinquan Chen, Chunlei Zheng, Liangliang Lou

**Affiliations:** ^1^Institute of Intelligent Information Processing, Taizhou University, Taizhou, Zhejiang, China; ^2^School of Science, Zhejiang University of Science and Technology, Hangzhou, Zhejiang, China; ^3^China United Network Communications Co., Ltd., Taizhou Branch, Taizhou, Zhejiang, China; ^4^Key Laboratory of Wireless Sensor Networks and Communications, Shanghai Institute of Microsystem and Information Technology, Shanghai, China

**Keywords:** human-vehicle recognition, channel status information, attention mechanism, depthwise separable convolution, wireless sensing

## Abstract

Wireless sensing-based human-vehicle recognition (WiHVR) methods have become a hot spot for research due to its non-invasiveness and cost-effective advantages. However, existing WiHVR methods shows limited performance and slow execution time on human-vehicle classification task. To address this issue, a lightweight wireless sensing attention-based deep learning model (LW-WADL) is proposed, which consists of a CBAM module and several depthwise separable convolution blocks in series. LW-WADL takes raw channel state information (CSI) as input, and extracts the advanced features of CSI by jointly using depthwise separable convolution and convolutional block attention mechanism (CBAM). Experimental results show that the proposed model achieves 96.26% accuracy on the constructed CSI-based dataset, and the model size is only 5.89% of the state of the art (SOTA) model. The results demonstrate that the proposed model achieves better performance on WiHVR tasks while reducing the model size compared to SOTA model.

## 1. Introduction

INTELLIGENT Traffic Systems (ITS) is an important part of smart city (Santos et al., 2018; Jin and Ma, 2019; Zhao et al., 2019; Choy et al., 2020; de Oliveira et al., 2020), providing reliable, safe, and convenient services for road users (e.g., cars, motorcycle, pedestrians, etc.). As the number of road users continues to increase, a large number of existing ITSs are approaching their limits. In order to improve the performance of ITSs and relieve traffic pressure, the measurement of traffic parameters including road user behavior has become a research hotspot (Jiang et al., 2021; Park et al., 2021; Zhao and Huang, 2021). Generally, the behavior of road users includes human-vehicle recognition (HVR), traffic flow statistics, vehicle speed, and direction measurement, etc. As the foundation of road user behavior detection, the accuracy of human-vehicle recognition determines the performance of traffic parameter measurement (Won et al., 2017; Sliwa et al., 2020).

With the rapid development of artificial intelligence and deep learning techniques, image-based HVR methods (Huang et al., 2020; Du et al., 2021) have been widely used in ITSs. Such HVR methods not only achieve excellent recognition performance, but also provide rich traffic image information for city managers. However, image-based HVR methods are susceptible to light so that their performance can be degraded rapidly in the case of low-light conditions such as night, cloudy, haze, etc. To alleviate the limitations of image-based HVR methods in low-light scenes, the microwave radar-based HVR method is proposed (Park et al., 2021; Singh et al., 2021; Tavanti et al., 2021). However, high-performance frequency-modulated continuous-wave (FMCW) radars come at a higher cost. In addition, the microwave radar has the problem of installation viewing angle, which leads to the high construction cost of the microwave radar-based HVR method.

Generally, the purpose of wireless sensing-based HVR (WiHVR) methods is to extract the energy information of surrounding wireless signals for target recognition. Since the propagation of wireless signals has no directionality, the WiHVR method does not have the problem with the above-mentioned viewing angle (Ma et al., 2019; Pan et al., 2019; Zhang et al., 2021). In recent years, the WiHVR methods based on extracted receiving signal strength (RSS) or channel state information (CSI) signatures from the wireless transceivers on 2.4 GHz band, such as Bluetooth (Sliwa et al., 2020; Wilby et al., 2020), ZigBee (Wang et al., 2017; Jiang et al., 2021), and WiFi (Chen et al., 2018; Wang F. et al., 2018), etc., have been widely employed to detect road users in ITSs.

### 1.1. CSI-based HVR methods

Wang W. et al. (2016) converted CSI signals to spectrograms, thereby describing human motion. Won et al. (2017) proposed a WiFi-based traffic monitoring system, in which the features of root mean square, median absolute deviation, mean, first quartile, and third quartile of the CSI signals were extracted, followed by a support vector machine for vehicle classification. Liu et al. (2017) showed a human motion detection method based on CSI phase difference. They discussed the situation of line of sight (LOS) and non-line of sight (NLOS). Arshad et al. (2018) proposed a WiFi-based device-free dangerous driving recognition system. This system extracted multi-domain features for both magnitude and phase of CSI signals. Wang J. et al. (2018) presented a new general device-free identification framework *via* empirical mode decomposition. They decomposed CSI signals into intrinsic mode functions (IMF) and extracted the time domain and frequency domain features from IMF components.

### 1.2. RSS-based HVR methods

Jiang et al. (2021) calculated the amplitude and mean information of RSS signals. They designed a HVR algorithm for WiHVR based on the calculated RSS features. Sliwa et al. (2020) provided a vehicle detection and classification method on the basis of the extracted RSS from transceivers on 2.4 GHz band. They used mean, minimum, standard deviation, and other characteristics of RSS signals to address the challenges of accuracy, robustness, and privacy. Abdelnasser et al. (2018) exploited a gesture recognition system in which the edge, frequency, and magnitude features of RSS signals were extracted for gesture recognition. Bhat et al. (2020) extracted the RSS power levels for human locomotion walking pattern recognition.

However, the above-mentioned WiHVR methods based on extracted RSS or CSI signatures from 2.4 GHz wireless transceivers like Bluetooth, ZigBee, and WiFi have the following drawbacks:

1) RSS is a coarse-grained signal, which leads to limited accuracy of HVR tasks based on RSS signals.
2) The effects of CSI or RSS on the performance of WiHVR in different application scenarios are not explored.

Recently, deep learning techniques (LeCun et al., 2015) consisting of a multi-layer network architecture have attracted much interest. One of the representative deep learning techniques is convolutional neural network (CNN) (Krizhevsky et al., 2012). Up to now, due to the powerful feature learning ability, CNNs have exhibited promising performance on various tasks such computer vision (Szegedy et al., 2016), speech signal processing (Zhang et al., 2017), natural language processing (Otter et al., 2020), and so on. However, few works have attempted to exploit the application of CNNs on WiHVR tasks.

To address the above-mentioned issues, this paper presents a novel WiHVR method based on the designed lightweight wireless sensing attention-based deep learning model (LW-WADL). Inspired by the recent-emerged convolutional block attention mechanism (Woo et al., 2018) (CBAM) and depthwise separable convolutions (Chollet, 2017), we propose a new deep model, which consists of a CBAM module and three depthwise separable convolution blocks in series to learn high-level features from preprocessing CSI signals for WiHVR. Compared with ordinary convolutions, depthwise separable convolutions have relatively low parameters and operations. Besides, we propose a novel CSI data enhancement method and a new subcarrier selection method. In particular, a new CSI-based dataset relates to road user behavior is constructed. In order to explore the effects of CSI on the performance of WiHVR in different application scenarios, the CSI dataset is divided into three taxonomies according to the number of categories, namely, two-category dataset, three-category dataset, and four-category dataset. Experimental results show that the accuracy of CSI-based methods decreases as the number of classification categories increases. For four-classification experiments, the proposed model achieves 96.26% accuracy and the model size is only 5.89% of the state of the art model.

To summarize, the main contributions of this paper are as follows:

1) This paper proposes a CSI data enhancement method, which preprocess the change trend of CSI data to one direction, thereby enhancing CSI data.
2) This paper provides a subcarrier selection method, which selects several subcarriers with large signal-to-noise ratios (SNR) as benchmarks and integrates them into a new CSI data.
3) This paper has proposed a lightweight wireless sensing attention-based deep learning model, and attempts to explore the effects of CSI on the performance of WiHVR in different application scenarios.

The remainder of this paper is organized as follows. Section “2. Preliminaries” introduces the CSI extraction and the theoretical analysis of WiHVR. Section “3. Proposed method” elaborates the proposed LW-WADL for WiHVR. Section “4. Experiment study” shows experimental results and analysis. Section “5. Conclusion and future work” gives the conclusions and future work.

## 2. Preliminaries

**FIGURE 1:**
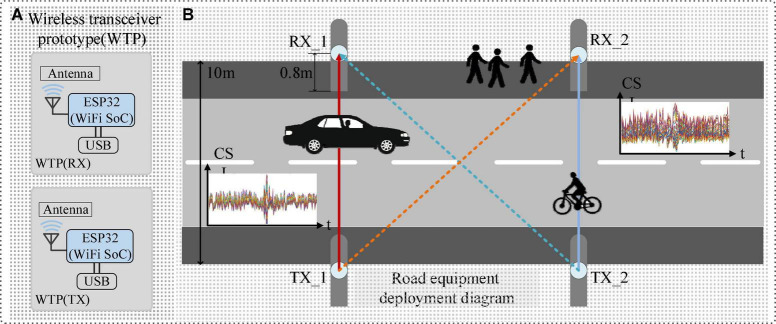
System architecture of the proposed WiHVR method. **(A)** Wireless transceiver prototype (WTP). **(B)** Road equipment deployment diagram.

This paper aims to establish a lightweight and efficient WiHVR method to explore the effects of CSI on the performance of WiHVR in different application scenarios. The system architecture of the proposed WiHVR method is shown in Figure 1.

From Figure 1, it can be found that wireless transceiver prototype (WTP) is built and placed on both sides of the road. The WTP is mastered by the ESP32 chip for generating and receiving wireless signals. Once a road user appears in the WTP sensing area, the CSI signal collected by the WTP will be attenuated due to the road user. Therefore, WTP can extract CSI signals related to the road user information.

### 2.1. CSI extraction

This paper uses the designed WTP to extract CSI data related to road users. CSI represents the fine-grained channel features of wireless communication links between transmitters and receivers based on orthogonal frequency division multiplexing (OFDM) technology. Besides, CSI describes the changes of phase and amplitude caused by multipath effect and transmission loss in wireless signal transmission. The CSI channel gain matrix is expressed as:


(1) $$M\,c\,s\,i=\left(\begin{array}{ccc} h_{11} & \ldots & h_{1\,n}\\ \vdots & \ddots & \vdots \\ h_{m\,1} & \cdots & h_{m\,n} \end{array}\right)$$

where *h*_*mn*_ represents the different subcarriers. *m* and *n*represent the transmitting and receiving antennas, respectively. Each sub-element *h*_*mn*_ represents:


(2) $$h_{m\,n}=||h_{m\,n}||\,e^{j\,\eta_{m\,n}}$$

where ||*h*_*mn*_|| is the amplitude of the sub-carrier *h*_*mn*_, and *e^j^*^η^_*mn*_ represent the phase of *h*_*mn*_. From Eqs 1, 2, it can be known that CSI is not a supersession of all subcarrier signals, it describes a multipath signal with more characteristics. In this case, the CSI extracted by WTP contains multiple subcarrier information. These subcarriers have different sensitivities to road users, so it is necessary to filter out the subcarriers with lower sensitivity. The specific method will be elaborated in Section “3. Proposed method.”

**FIGURE 2:**
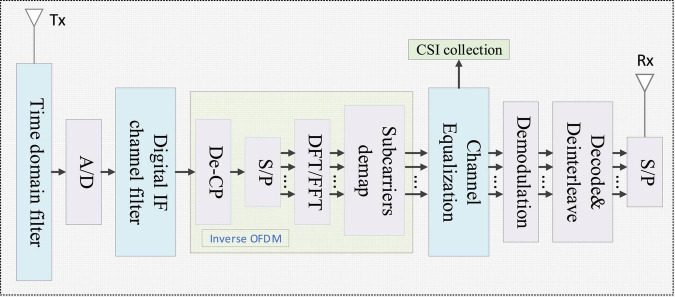
The specific process of CSI extraction.

The specific process of CSI signal extraction is shown in Figure 2. The acquisition of CSI signal needs to be operated by inverse OFDM. In order to eliminate inter-symbol interference and inter-channel interference, OFDM will use cyclic prefix (C/P), but this part is not real data, so this part needs to be removed in inverse OFDM. After that, it is necessary to convert the series signal to the parallel signal (S/P), and perform discrete Fourier transform (DFT) or fast Fourier transform (FFT) to obtain the required CSI signal.

### 2.2. Theoretical analysis of WiHVR

The idea of WiHVR is based on the fact that road users of existence and movement affect the wireless propagation paths. To understand the relation of road users movement with received CSI, the wireless propagation model should be first studied. In a typical wireless environment, there is one main path line-of-sight (LOS) and several reflected paths by the surroundings. As shown in Figure 1, if a road user is present in the WTP sensing area, it will cause multipath propagation of the wireless signal. In this case, according to the free space model, the received power by a receiver antenna which is separated from a radiating transmitter antenna by a distance _*d*_, is given by the Friis free space equation,


(3) $$P_{\text{r}}=\frac{P_{t}\,G_{t}\,G_{r}\,\lambda^{2}}{{\left(4\,\pi \right)}^{2}\,d^{2}}$$

where *P*_*r*_ and *P*_*t*_ are the receiving and transmitting power, respectively. *G*_*r*_ and *G*_*t*_ are the receiving and transmitting antenna gains, respectively. λ is the wavelength in meters. *d* is the distance between the transmitter and receiver in meters, that is, the propagation path length. When a road user exists in the wireless environment, several scattered paths are produced by road user. Those scattered power should also be added in the final received power.


(4) $$P_{\text{r}}=\frac{P_{t}\,G_{t}\,G_{r}\,\lambda^{2}}{{\left(4\,\pi \right)}^{2}\,\left(d^{2}+\delta^{2}\right)}$$

where δ is a brief representation of path length caused by road user. If a road user is static in the environment, *P*_*r*_ is almost stable. However, along with the move of a road user, the scattered paths change in a fast speed, resulting in the variance in received signal power.

According to Eq. 4, the differences in size and speed of road users lead to different attenuation of wireless signals. Hence, the CSI readings measured by the WTP prototype are various.

## 3. Proposed method

According to the above analysis, since the differences in the size and speed of humans or vehicles moving on the road, the attributes of energy attenuation caused by two targets are various. In this case, it is feasible to design a WiHVR method. To this end, a deep learning-based WiHVR method is proposed in purpose of analyzing the effects of CSI on the performance of WiHVR in different application scenarios.

### 3.1. System overview

**FIGURE 3:**
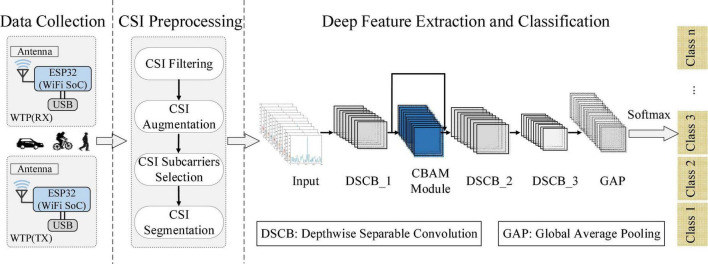
Overview of the proposed WiHVR based on a lightweight wireless sensing attention-based deep learning model (LW-WADL).

The overview of the proposed WiHVR based on a lightweight wireless sensing attention-based deep learning model (LW-WADL) is shown in Figure 3. The proposed WiHVR contains three key modules: Data collection, CSI preprocessing, and Deep feature extraction and classification. The data collection module consists of a pair of WTPs, both of which are made up of an ESP32 module, so as to collect CSI data of different road users in WTPs sensing area. The CSI preprocessing module includes CSI filtering, CSI augmentation, CSI subcarriers selection, and CSI segmentation. The core deep feature extraction and classification module, i.e., the proposed LW-WADL method consisting of a CBAM module and three depthwise separable convolution blocks, followed by a global average-pooling (GAP) layer for reducing computational complexity. In addition, GAP essentially is an average pooling operation which is intended to replace fully connected layers in classical CNNs. Thus, GAP is a special kind of average pooling where the sliding window of the average operation expands to the entire feature maps. Besides, after completing the final feature representations of the GAP layer, a _*C*_-class vector (_*C*_ is the number of categories) is output through the Softmax function.

### 3.2. Data collection

As shown in Figure 3, this paper captures the CSI data in space through the developed WTP. To extract CSI data, a threshold-based road user detection algorithm is exploited in this paper. The purpose of road user detection is to find out whether there are dynamic targets in the sensing area. According to the analysis in Section “2. Preliminaries,” it can be found that when there are no road users in the wireless environment, the CSI patterns stabilize around a reference value. Once a road user passes through the wireless environment, the amplitude of CSI patterns will drop sharply. Therefore, the presence of road users in the region of interest can be detected by the following threshold-based algorithm:


(5) $$X_{\det e\,c\,t\,i\,o\,n}\,\left[k+1\right]=$$

$$\left\{ \begin{array}{c} S\,t\,a\,t\,i\,c,\,X_{\det e\,c\,t\,i\,o\,n}\,\left[k\right]=D\,e\,t\,e\,c\,t\,e\,d\,\mathrm{and}\\ \prod_{n=k-W+1}^{k}sign\left(|x\left[n\right]-x_{s\,t\,a\,t\,i\,c}\left[n\right]|<T_{o\,b\,j\,e\,c\,t}\right)>0\\ D\,e\,t\,e\,c\,t\,e\,d,X_{\det e\,c\,t\,i\,o\,n}\,\left[k\right]=S\,t\,a\,t\,i\,c\,\mathrm{and}\\ \prod_{n=k-W+1}^{k}sign\left(|x\left[n\right]-x_{s\,t\,a\,t\,i\,c}\left[n\right]|\geq T_{o\,b\,j\,e\,c\,t}\right)>0 \end{array}\right.$$

where *x*[*n*](*n* > 0) represents the *n*-th CSI reading. *x*_*static*_[*n*] is the average of CSI readings when there are no road users in the wireless environment, namely, CSI baseline. *T*_*object*_ is the decision threshold to determine whether there is a road user (dBm). Here, *T*_*object*_ is set to 4 dBm in this work. *W* is the size of the judgment window, and is set to 50 when the sampling rate is 50 Hz. *X*_det⁡*ection*_[] is the object detection result. “Detected” indicates that there are road users in the range of interest, and otherwise “Static” denotes no road users.

Moreover, the environmental factors such as rain, fog, temperature, etc., can affect the CSI baseline *x*_*static*_[]. Thus, to improve the performance of the above-mentioned fluctuation detection algorithm, an adaptive baseline adjustment method is proposed, which can be calculated by:


(6) $$x_{s\,t\,a\,t\,i\,c}\,\left[n+1\right]=\left\{ \begin{array}{c} \beta \cdot x_{s\,t\,a\,t\,i\,c}\,\left[n\right]+\left(1-\beta \right)\cdot x\,\left[n+1\right],\\ X_{\det e\,c\,t\,i\,o\,n}\,\left[n\right]=X_{\det e\,c\,t\,i\,o\,n}\,\left[n+1\right]=S\,t\,a\,t\,i\,c\\ x_{s\,t\,a\,t\,i\,c}\,\left[n\right],o\,t\,h\,e\,r\,s \end{array}\right.$$

where _β_ is a correction factor with a value of 0.96 in this paper. It can be seen from Eq. 6 that the CSI baseline will be updated as long as there are no road users in the wireless environment, otherwise it will not be updated. Hence, the problem of CSI baseline drift caused by environmental factors can be solved efficiently, as well as the robustness of the fluctuation detection algorithm can be improved.

Finally, the CSI data extracted by WTP contains 52 subcarriers, and each subcarrier contains amplitude and phase information. In order to improve execution efficiency of LW-WADL, this paper converts the raw two-dimensional CSI data containing amplitude information into one-dimensional data. Then, one-dimensional CSI data containing road user behavior information will be sent to the second stage for data preprocessing.

### 3.3. CSI preprocessing

The CSI preprocessing module includes the following four steps: CSI filtering, CSI augmentation, CSI subcarriers selection, and CSI segmentation.

#### 3.3.1. CSI filtering

**FIGURE 4:**
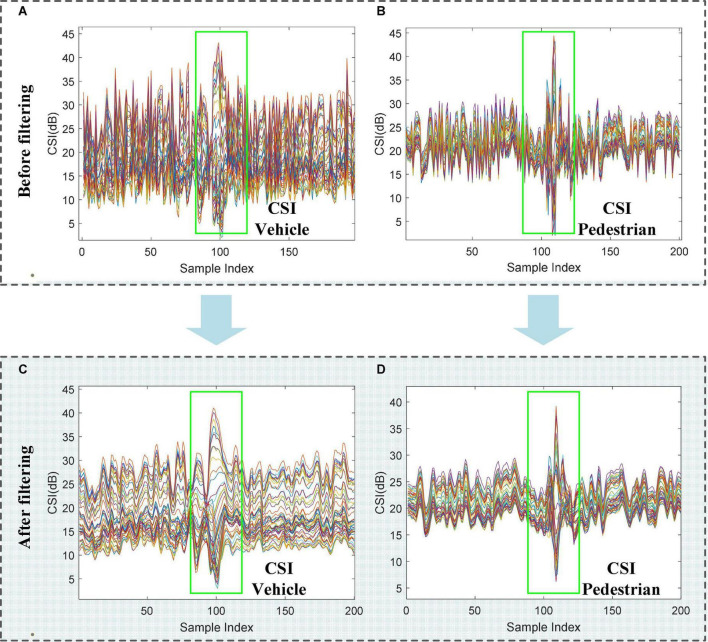
Raw CSI waveform vs. filtered waveform. **(A)** The raw CSI waveform of the vehicle. **(B)** The raw CSI waveform of the pedestrian. **(C)** The filtered CSI waveform of the vehicle. **(D)** The filtered CSI waveform of the pedestrian.

To guarantee the robustness of road users recognition, smoothing filtering is used to remove noise from the raw CSI data, as defined by:


(7) $$X_{f\,i\,l\,t\,e\,r}\,\left(n\right)=\frac{1}{N}\,\sum_{j=1}^{N-1}X_{r\,a\,w}\,\left(n-j\right)$$

where $X_{r\,a\,w}$ represent the raw CSI data, and *X*_*filter*_(*n*) is the average processed data and then the filter shift window size used is *N*, where is set to five. The raw CSI waveform vs. filtered waveform is shown in Figure 4. As can be seen from Figure 4, by applying moving average filter, the high-frequency noise has been removed from the CSI waveform without changing the trends of the waveform. The waveform changes of the filtered data (Figures 4C, D) are more pronounced than before filtering, thereby improving the efficiency and accuracy of road user detection.

#### 3.3.2. CSI augmentation

Channel state information augmentation aims to find a way to enhance the CSI features without changing the raw CSI features. According to the characteristics of the raw CSI signal waveform, this paper proposes a novel CSI data enhancement method. This method first calculates the average value of a set of CSI amplitude, and then takes the absolute value of the CSI amplitude which is smaller than the average value. In this way, the decay of the CSI amplitude is amplified, thereby enhancing CSI features. First, the baseline *X*_*base*_ of a set of CSI data needs to be calculated, which can be expressed as:


(8) $$X_{b\,a\,s\,e}=\frac{1}{I}\cdot \frac{1}{T}\,\sum_{i=1}^{I}\sum_{n=1}^{T}X_{c\,s\,i}\,\left(i,n\right)$$

where *i* represents the *i*-th CSI subcarrier, *n* represents the *n*-th sampling point of the *i*-th subcarrier. *I* represents the number of CSI subcarriers, which is 52 in this paper. T is the number of sampling points of a group of CSI data. The enhanced CSI data*X*_*csi*_*aug*_(*i*,*n*) can be obtained according to the CSI baseline *X*_*csi*_*base*_ :


(9) $$X_{c\,s\,i\,\_\,a\,u\,g}\,\left(i,n\right)=\left|X_{c\,s\,i}\,\left(i,n\right)-X_{c\,s\,i\,\_\,b\,a\,s\,e}\right|$$

where | | denotes the absolute value operation. According to the Eqs 8, 9, the enhanced CSI data can be obtained.

#### 3.3.3. CSI subcarriers selection

Although CSI augmentation have enhanced CSI features related to road users. In practical applications, different subcarriers of CSI have different sensitivities to road users, e.g., some subcarriers fluctuate greatly when encountering road users, while other subcarriers fluctuate less. Therefore, to further enhance CSI data, we design a raw CSI subcarrier selection method to remove subcarriers with low sensitivity in CSI data. In order to evaluate the sensitivity of CSI subcarriers, this paper calculates the SNR of the CSI data amplitude, as expressed by:


(10) $$S\,N\,R=10\,\mathrm{lg}\left|\frac{x_{p\,e\,a\,k}-x_{s\,t\,a\,t\,i\,c}}{n_{n\,o\,i\,s\,e}-x_{s\,t\,a\,t\,i\,c}}\right|$$

where *x*_*peak*_ is the peak value of CSI with respect to a road user. *x*_*static*_ is the average of CSI readings when there are no road users within a wireless environment. *n*_*noise*_ is the peak value of noise. According to Eq. 10, the SNR *X*_*csi*_*SNR*_(*n*) of all subcarriers in a set of CSI data is obtained:


(11) $$X_{c\,s\,i\,\_\,S\,N\,R}\,\left(n\right)=\left\{x_{S\,N\,R}\,\left(1\right),x_{S\,N\,R}\,\left(2\right),\ldots ,x_{S\,N\,R}\,\left(m\right),\ldots ,x_{S\,N\,R}\,\left(n\right)\right\}$$

where *x*_*SNR*_(*m*) represents the SNR value of the *m*-th subcarrier. For the convenience of calculation, it is assumed that *x*_*SNR*_(*n*)has been arranged in descending order of SNR, that is, {*x*_*SNR*_(1) > *x*_*SNR*_(2) > … > *x*_*SNR*_(*m*) > … > *x*_*SNR*_(*n*)}. According to Eq. 11, “m” subcarriers with larger SNR are selected, where “m” is defined as the CSI factor. The selection of the CSI factor “m” is discussed in detail in Section “4. Experiment study.” The mean of “m” subcarriers is calculated, which can be expressed as:


(12) $${\bar{X}}_{c\,s\,i\,\_\,a\,u\,g}\,\left(n\right)=\frac{1}{m}\,\sum_{n=1}^{m}X_{c\,s\,i\,\_\,a\,u\,g}\,\left(n\right)$$

**FIGURE 5:**
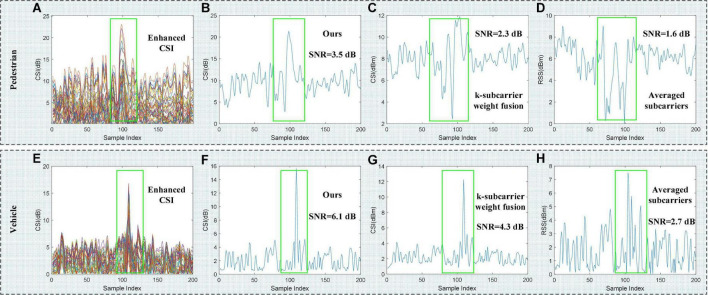
Different CSI subcarrier selection method. **(A,E)** Represent the enhanced raw CSI waveforms of the vehicle and pedestrian, respectively. **(B–D,F–H)** Represent the CSI waveforms of the vehicle and pedestrian generated by the three subcarrier selection methods, respectively.

To demonstrate the validity of Eq. 12, we compare our proposed method with k-subcarriers weight fusion (Kong et al., 2019) and average-subcarriers (Wang Y. et al., 2016), as shown in Figure 5. It can be seen that on a pedestrian and a vehicle CSI sample, our CSI subcarrier selection method performs best, the SNR of the CSI amplitude is 3.5 and 6.1 dB, respectively.

#### 3.3.4. CSI segmentation

The selected CSI data ${\bar{X}}_{c\,s\,i\,\_\,a\,u\,g}\,\left(n\right)$ containing multiple CSI features is split into certain segment-level sub-samples, each of which consists of one complete CSI feature of a road user. Specifically, the single CSI feature can be divided in terms of the local minimum in ${\bar{X}}_{c\,s\,i\,\_\,a\,u\,g}\,\left(n\right)$. These local minimums are defined as decision points. Specially, *x*_*d*_[*i*] represents the *i*-th decision point, *x*_*d*_[*i*] can be calculated as:


(13) $$x_{d}\,\left[i\right]=\min_{s\cdot \text{i-w}+1\leq n\leq s\cdot i}{\bar{X}}_{c\,s\,i\,\_\,a\,u\,g}\,\left(n\right),\,i=1,2,3,\ldots ,L$$

where *x*_*d*_[*i*] is the minimize value within the value range of ${\bar{X}}_{c\,s\,i\,\_\,a\,u\,g}\,\left(n\right)$. *L* is the number of decision points. *w* is the size of sliding window, and is set to 50, *s* is the step size of the window, and is set to 200. Additionally, the index of decision points in ${\bar{X}}_{c\,s\,i\,\_\,a\,u\,g}\,\left(n\right)$ is represented by *P*_*i*_ . According to Eq. 13 and *P*_*i*_, ${\bar{X}}_{c\,s\,i\,\_\,a\,u\,g}\,\left(n\right)$ can be divided into *L* segments. ${\tilde{x}}_{i}\,\left[n\right]$ is the _*i*_-th segment, which can be defined as:


(14) $${\tilde{x}}_{i}\,\left[n\right]=\left\{{\bar{X}}_{c\,s\,i\,\_\,a\,u\,g}\,\left[P_{i}-c_{0}\right],\ldots ,{\bar{X}}_{c\,s\,i\,\_\,a\,u\,g}\,\left[P_{i}\right],\ldots ,{\bar{X}}_{c\,s\,i\,\_\,a\,u\,g}\,\left[P_{i}+c_{0}\right]\right\}$$

where *c*_0_ is the slicing factor, and is set to 100. In this case, a new CSI dataset is developed. About 500 samples of four categories are included in the dataset: pedestrian, bicycle, motorcycle, and car.

### 3.4. Deep feature extraction and classification

According to the features of CSI signals containing time series features, as shown in Figure 3, a lightweight wireless sensing attention-based recognition algorithm, namely LW-WADL is proposed for deep feature learning from CSI features on HVR tasks. The proposed LW-WADL contains a CBAM module and three depthwise separable convolution modules, followed by a GAP layer, as described below.

#### 3.4.1. LW-WADL network structure

The overall network structure of the proposed LW-WADL is presented in Figure 3. LW-WADL involves of Three depthwise separable convolution blocks (DSCB_1, DSCB_2 and DSCB_3) in series. Then, in order to focus on learning the relevant information of feature maps while suppressing the irrelevant information, a CBAM module is concatenated after DSCB_1. The CBAM module can further improve the discriminating power of feature representations learned by DSCB_1. Finally, output features in DSCB_3 are achieved through a GAP layer.

#### 3.4.2. CBAM attention module

**FIGURE 6:**
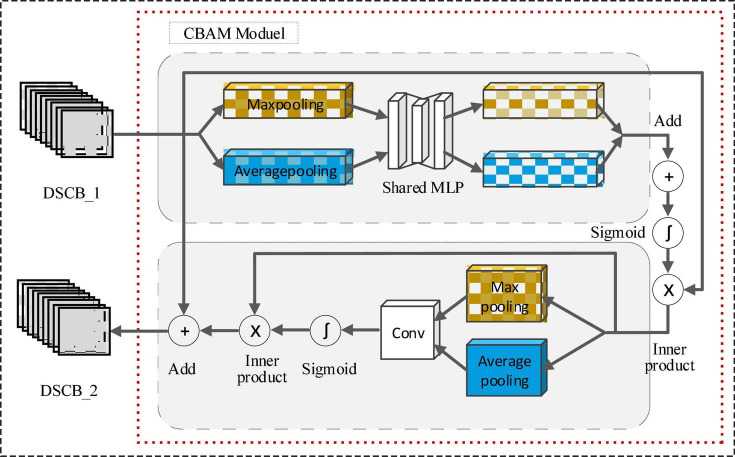
CBAM structure diagram.

The attention mechanism makes the model tend to pay attention to some information about the auxiliary classification in the feature map, while suppressing other useless information, thereby improving the classification ability of the model. The CBAM module consists of a channel attention module and a spatial attention module. The detailed structure is shown in Figure 6.

The channel attention module first performs maxpooling and average pooling based on the height and width of the DSCB_1 feature map to obtain two one-dimensional vectors. Then, it is input into the shared multi-layer perceptron (Shared MLP), and the corresponding elements of the output features of the MLP are summed point by point. The result is input into the Sigmoid activation function, and then the inner product operation is performed with the initial input feature map. The final output feature map is used as the input of the spatial attention module.

The spatial attention module performs maxpooling and average pooling based on the channel, and then uses the convolution (abbreviated as Conv) operation to merge the output features on the channel dimension. The merged features are input into a sigmoid activation function, then an inner product operation is performed on the obtained output features and the input of the spatial attention module. Finally, the output of the inner product operation is combined with the output of the DSCB_1 module to form the input features of the DSCB_2 module.

#### 3.4.3. Softmax classifier output

WiHVR is fundamentally a multi-classification task, so we choose the Softmax function to produce final classification results. Through the Softmax function, the output values of classifier can be converted into a probability distribution in the range [0, 1].

The cross-entropy loss function is implemented as the training objective function for LW-WADL:


(15) $$L_{l\,o\,s\,s}=-\sum_{i}{\hat{y}}_{i}\,\log\left(y_{i}\right)$$

where ${\hat{y}}_{i}=1$ if the class is *i*, otherwise ${\hat{y}}_{i}=0$. *y*_*i*_ represents the output of the LW-WADL model, the probability that the class is *i*. *L*_*loss*_ is a loss measure of the difference between two probability distributions.

## 4. Experiment study

### 4.1. Experiment setup

**FIGURE 7:**
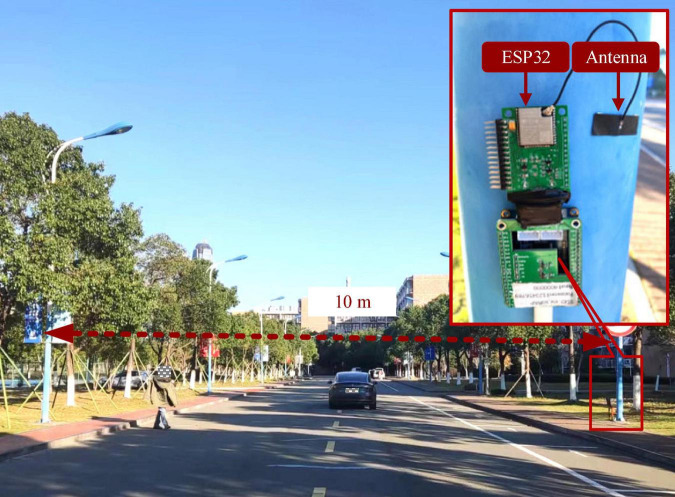
Experimental scenarios and WTP installation details.

As can be seen from Figure 7, the proposed WTP prototype contains two main components: antenna, ESP32. ESP32 is a WiFi SoC working at a frequency of 2.4 GHz. In the experiment, the two WTP prototypes were installed on both sides of a road with a width of 10 m, and antenna heights is set to 1 m. In addition, for training LW-WADL models, the Adam optimizer with a learning rate of 0.0001 is used. The batch-size is 16 and the maximum of epochs is 200. Besides, to explore the effects of CSI on the performance of WiHVR in different application scenarios, the developed CSI dataset is divided into three taxonomies according to the number of categories, namely, two-category dataset, three-category dataset and four-category dataset. Finally, 80% of the data in the dataset is used as the training set, while the rest is used for testing.

### 4.2. Evaluation indicators

The performance of the designed LW-WADL is evaluated by three typical metrics such as “Accuracy,” “Recall,” and “Precision.” For the computational complexity analysis of deep learning methods, two well-known computational indicators, the network parameters (abbreviated as param.) and floating-point operations (FLOPs) are employed. Specifically, “Accuracy” is the ratio of all correct predictions to the whole number of predictions. “Precision” is the ratio of correct predictions with positive values to total predictions with positive values. “Recall” is the ratio of predicted positives to the total number of actual positives. They are defined as:


(16) $$A\,c\,c\,u\,r\,a\,c\,y=\frac{T\,P+T\,N}{T\,P+T\,N+F\,N+T\,N}$$

(17) $$P\,r\,e\,c\,i\,s\,i\,o\,n=\frac{T\,P}{T\,P+F\,P}$$

(18) $$R\,e\,c\,a\,l\,l=\frac{T\,P}{T\,P+F\,N}$$

where *TP* denotes the number of true positive samples classified as positive. *FP* denotes the number of true negative samples classified as positive. *FN* denotes the number of true positive samples classified as negative. *TN* denotes the number of true negative samples classified as negative.

### 4.3. Comparison of different methods

To verify the effectiveness of our LW-WADL for WiHVR, we adopt the developed CSI four-category dataset, so as to compare the performance of various deep learning models on WiHVRs tasks. Table 1 present a performance comparison of four methods, including full convolutional network (FCN) (Long et al., 2015), DeepWiTraffic (Won et al., 2019), and deep residual network (ResNet) (He et al., 2016). Among them, FCN and ResNet are used as baseline methods to provide benchmarking performance, whereas DeepWiTraffic is used as comparing work. Besides, FCN is composed of three convolutional layers. ResNet contains three residual blocks. DeepWiTraffic contains two convolutional layers and two max pooling layers. Experimental results are shown in Table 1.

**TABLE 1:** Performance comparison of different methods on four-category dataset.

Method	Accuracy (%)	Precision (%)	Recall (%)	Param (M)	FLOPs (M)	Testing time(s)
FCN	93.46%	93.34%	93.46%	1.6451	3.2888	0.2019
DeepWiTraffic	94.59%	94.45%	94.59%	0.2090	0.4176	0.1398
ResNet	94.39%	95.28%	94.39%	0.7427	1.5047	0.2982
Ours	**96.26%**	**96.23%**	**96.16%**	**0.0123**	**0.0282**	**0.0575**

The bold values represent the indicator with the best performance, that is, the highest Accuracy, Precision, and Recall as well as the lowest Param, FLOPs, and Testing time.

From Table 1, it can be found that the designed deep learning model has the highest classification performance with an accuracy of 96.26%, a precision of 96.23%, and a recall of 96.16%. Compared with DeepWitraffic, our model not only makes an improvement of 1.67%, but also exhibits much lower computational complexity in which 93.25% of the parameters (Param, FLOPs) can be reduced. Moreover, the test time is just 0.0575 s, which is much less than DeepWitraffic. This shows that our model is a lightweight model. Additionally, compared with FCN and ResNet, our method yields an accuracy improvement of 2.8 and 1.87%.

### 4.4. Selection of CSI factor m

**FIGURE 8:**
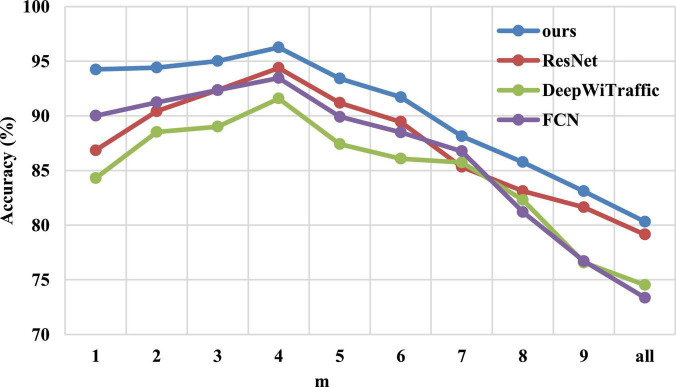
The classification accuracy of different CSI factors “m” on the CSI four-category dataset.

A set of experiments are designed to investigate the effect of CSI factor “m” on the accuracy of WiHVR. Figure 8 shows the accuracy of the WiHVR for different “m,” where “all” represents the maximum “m.” Experiments are performed on the CSI four-category dataset. Each CSI factor “m” corresponds to a CSI four-category dataset, and these datasets are identical except for the CSI factor “m.” To make the results more reliable, the ResNet, DeepWiTraffic, FCN, and our model are used. The experimental results are shown in Figure 8.

As shown in Figure 8, with the increase of “m,” both the ResNet, DeepWiTraffic, FCN, and ours model show a trend of increasing first and then decreasing, reaching the highest accuracy of 94.39, 94.59, 93.46, and 96.26% when “m” is 4, respectively. It can be found that only one subcarrier with the highest SNR or the average of all subcarriers cannot obtain the best HVR performance. This is because the sensitivity of different CSI subcarriers varies greatly. Some subcarriers are less sensitive, while some subcarriers with higher sensitivity are too sensitive to environmental changes, resulting in reduced recognition ability.

### 4.5. Comparison of CSI subcarrier selection methods

To verify the performance of the proposed CSI subcarrier selection method, we conduct comparative experiments on the CSI four-category dataset. Recently-merged CSI subcarrier selection methods such as k-subcarrier weight fusion (Kong et al., 2019) and averaged subcarriers (Wang Y. et al., 2016) are used for comparative experiments. The recognition accuracy of FCN, DeepWiTraffic, ResNet, and our LW-WADL under different CSI subcarrier selection methods are shown in Table 2.

**TABLE 2:** Accuracy of different subcarrier selection methods.

CSI subcarrier selection methods	FCN	DeepWiTraffic	ResNet	LW-WADL
k-subcarrier weight fusion	89.17%	90.86%	90.77%	93.01%
Averaged subcarriers	82.09%	87.89%	83.36%	90.53%
Ours	**93.46%**	**94.59%**	**94.39%**	**96.26%**

The bold values represent that our subcarrier selection method achieves the highest accuracy among the four compared deep learning models.

As shown in Table 2, the four used models perform best under our CSI subcarrier selection method, and the accuracy of HVR is 93.46, 94.59, 94.39, and 96.26%, respectively. The results show the superiority of our CSI subcarrier selection method, which is consistent with the conclusion drawn in Figure 5. In addition, the k-subcarrier weight fusion and averaged subcarriers methods will not remove those CSI subcarriers with too low or too high sensitivity, which may have a negative impact on CSI waveform. In this case, the accuracy of the above two methods in Table 2 is lower than that of our proposed method.

### 4.6. Performance evaluation of different classification tasks

To explore the performance of CSI signals on different classification tasks, three groups of experiments are set up, namely two-classification tasks, three-classification tasks, and four-classification tasks. Each group of experiments selects four methods to test, FCN, DeepWiTraffic, ResNet, and our LW-WADL, respectively, so that the results are more credible. The experimental results are shown in Table 3.

**TABLE 3:** Performance evaluation of different classification tasks.

Tasks	FCN	DeepWiTraffic	ResNet	LW-WADL
2	100%	100%	100%	**100%**
3	95.12%	96.86%	96.21%	**97.96%**
4	93.46%	94.59%	94.39%	**96.26%**

The bold values indicate that the designed deep learning model (LW-WADL) outperforms on different classification tasks.

The results of Table 3 shows that the compared methods perform best and the same on the two-classification task, and the accuracy of HVR reaches 100%. However, with the increase of road user categories, the classification accuracy of the used four methods decrease. For three-classification task, the classification accuracy of four methods are 95.12, 96.86, 96.21, and 97.96%, respectively. For four-classification task, the classification accuracy of four methods is 1.66, 2.27, 1.82, and 1.73% lower than three-classification task. Overall, our method achieves more than 96% accuracy in different classification tasks.

### 4.7. CSI confusion matrices

**FIGURE 9:**
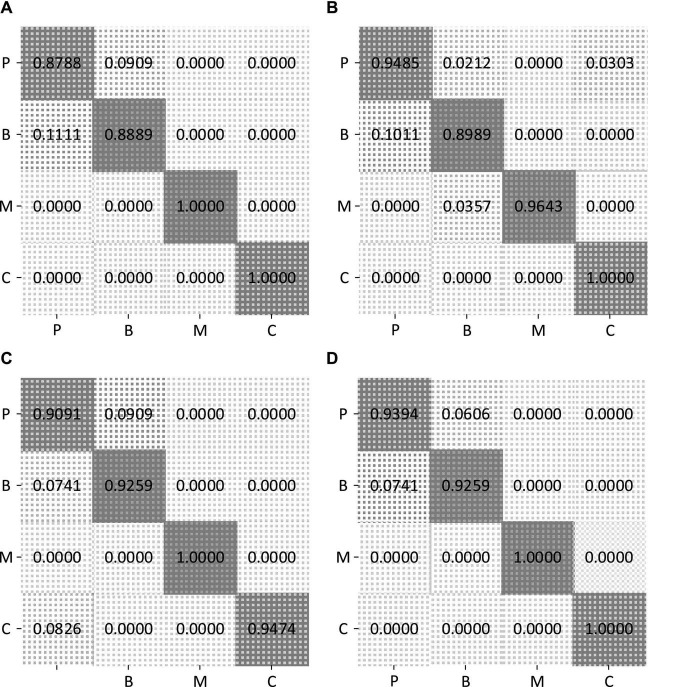
Confusion matrices of four methods on CSI four-classification dataset. **(A)** CSI confusion matrix of FCN. **(B)** CSI confusion matrix of DeepWiTraffic. **(C)** CSI confusion matrix of ResNet. **(D)** CSI confusion matrix of ours. P, pedestrian; B, bicycle; M, motorcycle; C: car.

To further display the recognition accuracy for each class of road users, Figure 9 shows confusion matrices of the classification results, when FCN, DeepWiTraffic, ResNet, and our LW-WADL methods obtain 93.46, 94.59, 94.39, and 96.26% accuracy. As shown in Figure 9, the “car” category is well-recognized for most of used methods. Among all the models, only ResNet incorrectly classify the “car” as the “pedestrian.” This may be attributed to the fact that a car usually has a much larger volume than other road users. As a result, the attenuation of CSI readings caused by cars is quite different from those cases caused by other road users. For all used models, a major part of the error arises from misclassifying “pedestrian” as “bicycle.” This reveals that the group, i.e., “pedestrian” vs. “bicycle” is easily confused with each other. This phenomenon is hinted by the overlap among some real-world road user shapes.

## 5. Conclusion and future work

This paper has proposed a lightweight wireless sensing attention-based deep learning model (LW-WADL). In order to evaluate the classification ability of LW-WADL, three CSI-based datasets are established, namely two-category dataset, three-category dataset. and four-category dataset. The experimental results on the developed dataset show that the classification accuracy of LW-WADL decreases with the increase of road user categories, but it is higher than 96%. In addition, this paper provides a novel CSI subcarrier selection method, which calculates the SNR of all subcarriers and selects the first four subcarriers with larger SNR for fusion. Besides, a new CSI data enhancement method is exploited to preprocess the change trend of CSI data to one direction, thereby enhancing CSI data.

In future, the performance of other advanced deep learning-based WiHVR methods will be investigated. It is also significant to explore the human-vehicle recognition task based on multiple sets of WTPs. Additionally, it is meaningful to explore the applications of the proposed methods in real scenarios such as multi-lane roads.

## Data availability statement

The raw data supporting the conclusions of this article will be made available by the authors, without undue reservation.

## Author contributions

MS was responsible for the writing of the manuscript and some experiments. RZ was responsible for some experiments of the manuscript. XC was responsible for the data collection and processing of the manuscript. CZ was responsible for the revision of the manuscript. LL provided the fund support for this project. All authors contributed to the article and approved the submitted version.
